# Monolithic Zirconia Versus Porcelain‐Fused‐to‐Metal Implant‐Supported Fixed Dental Prostheses in the Posterior Region: 3‐Year Results of a Randomized Controlled Clinical Trial

**DOI:** 10.1111/clr.70160

**Published:** 2026-08-21

**Authors:** Aspasia Pachiou, Raphael Singh, Ronald E. Jung, Marc Balmer, Goran I. Benic, Alexis Ioannidis

**Affiliations:** ^1^ Clinic of Reconstructive Dentistry, Center for Dental Medicine University of Zurich Zurich Switzerland

**Keywords:** dental implant, dental implant therapy, implant‐supported dental prosthesis

## Abstract

**Objectives:**

To compare the clinical, biological, and technical outcomes of posterior three‐unit implant‐supported fixed dental prostheses (iFDPs) fabricated from monolithic zirconia (MonoZR) or porcelain‐fused‐to‐metal (PFM).

**Materials and Methods:**

In this prospective randomized controlled trial, partially edentulous patients requiring a posterior three‐unit iFDP supported by two implants were randomly assigned to MonoZR or PFM iFDPs. The primary outcome was ceramic fracture. Secondary outcomes included prosthesis survival, complication‐free survival, marginal bone level (MBL) changes, peri‐implant clinical parameters, and USPHS ratings. Follow‐up examinations were performed at 1 and 3 years. Statistical analyses included Kaplan–Meier estimates, Fisher's exact test, and linear mixed‐effects models (*α* = 0.05).

**Results:**

Fifty‐five patients received iFDPs at baseline (MonoZR: *n* = 28; PFM: *n* = 27), and 45 completed the 3‐year follow‐up. Two prosthesis failures occurred, one in each group, both associated with implant loss, with no difference in survival between groups (*p* = 1.00). Veneering ceramic chipping occurred only in the PFM group (3/23; 13%) and not in the MonoZR group (0/22) (*p* = 0.11). Complication‐free survival, peri‐implant clinical parameters, and MBL changes did not differ significantly between groups (*p* > 0.05). Plaque index and bleeding on probing increased over time, while probing depth and MBL remained stable. Both groups showed high clinical acceptability according to USPHS criteria.

**Conclusions:**

Both MonoZR and PFM posterior three‐unit iFDPs demonstrated high survival and favorable biological outcomes after 3 years. MonoZR showed no chipping events and may represent a promising alternative to PFM for posterior implant‐supported restorations.

**Trial Registration:** German Clinical Trials Register: DRKS00011039

## 1 Introduction

The selection of restorative materials for Implant‐Supported Fixed Dental Prostheses (iFDPs) plays a pivotal role in determining mechanical stability, biological integration, and long‐term clinical success (Strasding et al. 2024). For decades, Porcelain‐Fused‐to‐Metal (PFM) restorations have been regarded as a reliable reference standard for multi‐unit iFDPs, primarily due to their well‐documented and predictable performance (Sailer, Balmer, et al. 2018). However, biological and technical complications remain frequent, with systematic reviews reporting considerable cumulative complication rates over a five‐year period, and veneering ceramic chipping representing the most common technical failure (Pjetursson et al. 2012; Sailer, Balmer, et al. 2018; Rabel et al. 2018). Such failures often require corrective interventions and may compromise long‐term treatment outcomes.

In response to these challenges, zirconia‐based restorations have gained widespread adoption in implant prosthodontics (Pjetursson et al. 2022). Owing to their high strength, favorable biocompatibility, and compatibility with digital manufacturing techniques, zirconia materials are increasingly utilized for posterior restorations (Rabel et al. 2018). Conventionally, these restorations have been fabricated as bilayer systems, consisting of a zirconia substructure covered by a veneering ceramic. While this design can provide satisfactory esthetic results, it remains prone to cohesive and adhesive failures within the veneering layer, particularly under functional loading conditions (Ioannidis et al. 2026; Sailer, Balmer, et al. 2018; Sailer, Strasding, et al. 2018). This limitation is especially relevant in posterior regions, where occlusal forces are substantially higher (Koenig et al. 2013).

The introduction of monolithic zirconia has aimed to overcome these shortcomings by eliminating the veneering interface altogether (Cheng et al. 2019; Pjetursson et al. 2021). Improvements in material composition have enabled the production of full‐contour restorations that combine mechanical robustness with high esthetic properties. From a biomechanical perspective, monolithic zirconia demonstrates high fracture resistance, with laboratory studies reporting fracture loads that exceed typical masticatory forces in the posterior dentition (Johansson et al. 2014). Early clinical investigations, primarily focusing on single crowns and short‐span restorations, have reported encouraging outcomes (Cheng et al. 2018; Zumstein et al. 2023; Hjerppe et al. 2025). Nevertheless, data on multi‐unit implant‐supported applications remain limited, particularly with respect to longer‐term clinical performance.

Although a substantial body of literature exists for tooth‐supported prostheses (Romandini et al. 2026), evidence directly comparing restorative materials in implant‐supported multi‐unit frameworks is still scarce. In particular, Randomized Controlled Trials (RCTs) evaluating monolithic zirconia against established PFM restorations for posterior three‐unit iFDPs are lacking (Sailer, Balmer, et al. 2018; Kim et al. 2023). This absence of high‐quality comparative data restricts the ability to draw definitive conclusions regarding the relative benefits and risks of these material concepts in clinical practice.

Therefore, the present superiority RCT was designed to compare the clinical performance of three‐unit monolithic zirconia and conventional PFM iFDPs. The objective was to assess whether the monolithic approach is associated with a reduced incidence of ceramic fractures while maintaining comparable biological outcomes and prosthetic survival. The current report presents the 3‐year follow‐up results of this ongoing trial, providing insight into the mid‐term clinical performance of these two restorative strategies.

## 2 Material and Methods

### 2.1 Study Design

This investigation was conducted as a prospective, superiority RCT with two parallel groups at the Clinic of Reconstructive Dentistry, University of Zurich, Switzerland. Written informed consent was obtained from all participants prior to study inclusion. Ethical approval was granted by the local ethics committee of the Canton of Zurich, Switzerland (Ref. BASEC‐Nr. 2016‐00840), and the study was performed in accordance with the Declaration of Helsinki (1975, revised in 2013). The trial was registered in the German Clinical Trials Register (DRKS; No. DRKS00011039). Reporting of the trial followed the CONSORT guidelines for parallel‐group randomized studies (see Supporting Information).

### 2.2 Study Population

Patients presenting with partial edentulism and requiring a three‐unit iFDP in a posterior region of the maxilla or mandible were considered eligible for inclusion. All participants were invited to take part in the study and provided written informed consent prior to enrollment.

Inclusion criteria were as follows:

- Written informed consent.
- Age > 18 years.
- Indication for a three‐unit iFDP supported by one implant in the premolar region and one in the molar region (first premolar–first molar or second premolar–second molar).
- Presence of at least one opposing tooth.
- Implant positioning allowing for a screw‐retained restoration.

Exclusion criteria comprised:

- Pregnancy at the time of enrollment.
- Known or suspected non‐compliance, including drug or alcohol abuse.
- Inability to adhere to study procedures (e.g., due to language barriers, psychological disorders, or cognitive impairment).
- Smoking > 15 cigarettes per day.
- Poor oral hygiene (Plaque Index > 30%).

Participants were excluded if implant positioning did not permit screw‐retained prosthetic reconstruction. In such cases, replacement was performed if withdrawal occurred prior to iFDP insertion to maintain the intended sample size. The first participant was enrolled in January 2017 and the final participant in January 2021. Recruitment continued until the predefined sample size was reached.

### 2.3 Sample Size Calculation

Sample size estimation was performed using statistical software (G*Power; Heinrich Heine University, Düsseldorf, Germany) (Faul et al. 2007). Based on previous reports, the most frequent technical complication of metal–ceramic iFDPs is veneering ceramic fracture, with an estimated 3‐year incidence of approximately 34% (Heintze and Rousson 2010). It was hypothesized that monolithic zirconia restorations would provide superior performance outcomes, that is reducing this rate by up to 90%. Accordingly, a power analysis using Fisher's exact test was conducted. To detect a difference between 35% and 5% with a statistical power of 80% and a two‐sided alpha level of 0.05, a total of 25 patients per group (*n* = 50) was required. To compensate for potential dropouts, a total sample size of 52 patients was planned. Due to patient replacement during the treatment phase and continued recruitment, 55 patients ultimately received iFDPs.

### 2.4 Randomization and Allocation Concealment

Participants were allocated to one of the two treatment groups using a computer‐generated randomization sequence. Block randomization (block size = 4) with a 1:1 allocation ratio was applied to ensure balanced group sizes. In patients presenting with more than one eligible site, a single site was randomly selected using a die‐roll method.

Allocation concealment was maintained via sequentially numbered, opaque, sealed envelopes, which were opened only immediately prior to impression taking.

The treatment groups were defined as follows:

- *Test group (MonoZR):* monolithic iFDPs fabricated from third‐generation zirconia (DD Bio ZX^2^ 98; Dental Direkt, Spenge, Germany), adhesively bonded to titanium base abutments (Straumann Variobase for Bridge, Institut Straumann AG, Basel, Switzerland) using Panavia 21 OP cement (Kuraray Noritake Dental Inc., Tokyo, Japan).
- *Control group (PFM):* porcelain‐fused‐to‐metal iFDPs consisting of cast gold abutments (RN synOcta gold abutment; Institut Straumann AG, Basel Switzerland) fabricated from a high‐noble alloy (V‐Classic; Cendres+Métaux SA, Biel/Bienne, Switzerland) and veneered with feldspathic ceramic.

Patients who discontinued participation after iFDP placement (baseline) were not replaced. Blinding of outcome assessors was not feasible due to the distinct material characteristics of the restorations.

### 2.5 Implant Placement

All surgical procedures were performed by experienced clinicians within a university clinical setting following a standardized protocol. Prior to study initiation, all operators underwent calibration to ensure consistency in case selection, surgical procedures, and outcome assessment.

Implant placement was carried out according to Type 2, Type 3, or Type 4 timing protocols based on the ITI classification (Chen et al. 2004; Gallucci et al. 2018). Preoperative medication included administration of a nonsteroidal anti‐inflammatory drug (ibuprofen 400 mg). All interventions were performed under local anesthesia. A mid‐crestal incision was made, with vertical releasing incisions where indicated, followed by elevation of full‐thickness mucoperiosteal flaps.

Osteotomies were prepared according to the manufacturer's guidelines (Institut Straumann AG, Basel, Switzerland). The following implants were placed:

- *Mesial position:* 3.3‐mm diameter bone‐level implant (Straumann Bone Level, Roxolid, SLActive).
- *Distal position:* 4.1‐mm diameter soft‐tissue‐level implant (Straumann Tissue Level, Roxolid, SLActive).

A 3.3‐mm bone‐level implant was placed in the mesial (premolar) position and a 4.1‐mm tissue‐level implant in the distal (molar) position to accommodate the anatomical and prosthetic requirements of the respective sites while maintaining a standardized restorative protocol.

Primary implant stability was assessed clinically. In cases presenting with peri‐implant defects (dehiscences, fenestrations, infrabony defects > 0.5 mm, or thin buccal plates), guided bone regeneration was performed using deproteinized bovine bone mineral (Bio‐Oss; Geistlich Pharma AG, Wolhusen, Switzerland) in combination with a native collagen membrane (Bio‐Gide; Geistlich Pharma AG). Membranes were stabilized with resorbable pins when necessary.

Following implant placement, cover screws were inserted, and flaps were sutured using non‐resorbable ePTFE monofilament sutures (Gore‐Tex; W.L. Gore & Associates, Flagstaff, AZ, USA). Healing was performed either transmucosally or in a submerged manner depending on primary stability.

Postoperative care included chlorhexidine rinses (0.2%) twice daily and analgesics (ibuprofen 400 mg) as needed. According to the protocol of the clinic, neither preoperative antibiotic prophylaxis nor postoperative antibiotics were routinely prescribed for implant placement alone. Systemic antibiotics were prescribed only in cases requiring simultaneous bone augmentation. In these cases, postoperative Co‐Amoxicillin 625 mg was prescribed according to the protocol of the clinic. Sutures were removed after 7–10 days.

### 2.6 Prosthetic Procedures

In cases of submerged healing, second‐stage surgery was performed after 2–4 months under local anesthesia to place corresponding Straumann healing abutments (Straumann AG, Basel, Switzerland), including NC healing abutments for Bone Level implants and RN healing components for Tissue Level implants. Eligibility for screw‐retained restorations was reassessed prior to prosthetic procedures.

No provisional restorations were used. Final impressions were obtained either conventionally using a polyether material (Permadyne, 3 M ESPE, Seefeld, Germany) or digitally using an intraoral scanner (TRIOS 3; 3Shape, Copenhagen, Denmark).

Definitive iFDPs (MonoZR or PFM) were delivered 4–6 months following implant placement. All restorations were screw‐retained with a torque of 35 Ncm. Screw access channels were sealed using polytetrafluoroethylene (PTFE) tape and composite resin (Filtek Universal Restorative, 3 M ESPE, St. Paul, MN, USA). Occlusion was adjusted to achieve even and simultaneous centric contacts while avoiding premature contacts and excursive interferences. iFDPs were adjusted using a double‐shimstock protocol to provide slightly lighter occlusal contacts than the adjacent natural dentition, with one shimstock held on the implant‐supported restoration while the second could be withdrawn with slight resistance. Following occlusal adjustment, all modified ceramic surfaces were finished and polished using dedicated ceramic polishing instruments.

Baseline was defined as the time of iFDP insertion. Patients were scheduled for follow‐up examinations at 1 and 3 years post‐insertion. Participants missing baseline assessments but attending follow‐up visits were included where appropriate; however, they were excluded from analyses requiring longitudinal comparisons.

### 2.7 Outcome Variables

The primary outcome of the study was the incidence of ceramic fractures in the iFDPs, assessed according to modified United States Public Health Service (USPHS) criteria and considered an indicator of prosthetic success.

Secondary outcomes included implant survival, marginal bone level (MBL) changes evaluated on standardized periapical radiographs, clinical peri‐implant parameters (Probing Depth (PD), Plaque Index (PI) and Bleeding on Probing (BoP)), and the occurrence of biological and technical complications. Outcome measures were selected in accordance with the Implant Dentistry Core Outcome Set and Measurement (ID‐COSM) international consensus, encompassing surgical morbidity, peri‐implant tissue health, adverse events, complication‐free survival, and patient‐reported outcomes such as satisfaction and comfort.

### 2.8 Implant Survival

Implant survival was assessed over a 3‐year follow‐up period, with baseline defined as the time of iFDP insertion. An implant was classified as surviving if it remained in situ during the observation period. Clinical stability of implants and prostheses was additionally evaluated using manual mechanical testing. Stability of both implants and prostheses was evaluated using mechanical testing with manual instrumentation.

### 2.9 Radiographic Analysis

Standardized periapical radiographs were obtained immediately after implant placement, at the time of definitive iFDP insertion (baseline), and at the 3‐year follow‐up.

Radiographs were acquired using individualized holders to ensure reproducibility, with the film positioned parallel to the implant axis and the X‐ray beam oriented perpendicular to the implant. All images were digitized and analyzed using ImageJ software (version 1.43; National Institutes of Health, Bethesda, MD, USA).

Radiographic assessment was performed by a calibrated examiner blinded to the treatment allocation and study objectives. MBL was measured on the mesial and distal aspects of each implant as the distance from the implant shoulder to the first bone‐to‐implant contact. Calibration of measurements was performed using the known inter‐thread distance of the implants.

Mesial and distal values were averaged to obtain a single MBL value per implant. Changes in MBL were calculated from baseline (iFDP insertion) to the 3‐year follow‐up. Negative values indicated marginal bone loss, whereas positive values indicated bone gain.

### 2.10 Clinical Parameters

At the 3‐year follow‐up, clinical peri‐implant parameters were assessed, including PI (O'Leary et al. 1972), BoP (Ainamo and Bay 1975), and PD, each recorded at six sites per implant.

### 2.11 Complications and Adverse Events

All Adverse Events (AEs), as well as biological and technical complications, were recorded throughout the follow‐up period.

Biological complications included implant mobility, persistent subjective complaints, continuous peri‐implant radiolucency, and peri‐implant infection with suppuration. Peri‐implantitis was defined as marginal bone loss exceeding 2 mm in combination with positive BoP and the availability of baseline radiographs (Berglundh et al. 2018).

Technical complications comprised implant fracture, abutment fracture, veneering ceramic fracture, loosening of the abutment screw, and fracture of the abutment screw. In cases of complications, appropriate interventions were performed, and patients were followed until resolution.

### 2.12 Prosthetic Evaluation

Clinical performance of the iFDPs was assessed at the 3‐year follow‐up using the USPHS evaluation system. Examinations were conducted by calibrated clinicians according to predefined criteria.

All USPHS categories were recorded; however, selected clinically relevant parameters were included in the present analysis, including patient satisfaction (USPHS 1), veneering fracture (USPHS 4), and occlusal wear (USPHS 13). Outcomes were categorized as Alpha, Bravo, Charlie, or Delta.

### 2.13 Statistical Analysis

Statistical analyses were performed using R statistical software (R Foundation for Statistical Computing, Vienna, Austria). Statistical significance was set at *α* = 0.05, and all tests were two‐sided. Descriptive statistics were calculated for all variables. Continuous data are presented as mean ± standard deviation (SD), while categorical variables are reported as absolute and relative frequencies.

Prosthesis survival and complication‐free survival were analyzed using Kaplan–Meier estimates and compared between groups using the log‐rank test. Events were defined as prosthesis loss for survival analysis and as the occurrence of any biological or technical complication for complication‐free survival. The incidence of veneering material chipping between groups was compared using Fisher's exact test because of the low number of events.

Longitudinal changes in peri‐implant clinical parameters (PI, BoP, and PD) and MBL were analyzed using linear mixed‐effects models. For peri‐implant clinical parameters, group, time, and their interaction were included as fixed effects. For MBL, group, time, implant site (mesial/distal), and their interactions were included as fixed effects. Patient ID was modeled as a random intercept to account for repeated measurements within participants. A more complex random‐effects structure including random slopes for time was explored but resulted in a singular fit; therefore, the more parsimonious random‐intercept model was retained. An additional mixed‐effects model including bone augmentation procedures (yes/no) as a fixed effect was performed to evaluate their influence on MBL.

USPHS criteria were recorded according to the Alpha–Bravo–Charlie–Delta classification and summarized descriptively as frequency distributions. Between‐group comparisons at each follow‐up time point were performed using Fisher's exact test or Pearson's chi‐square test, as appropriate. Given the low frequency of non‐Alpha ratings, this approach was considered more appropriate than mixed‐effects logistic regression. Outcomes were additionally dichotomized into clinically acceptable (Alpha/Bravo) and unacceptable (Charlie/Delta) categories for clinical interpretation.

Model assumptions were assessed by visual inspection of residuals and Q–Q plots, evaluation of homoscedasticity, and graphical assessment of influential observations. No major violations were detected. All analyses were conducted according to the intention‐to‐treat principle, as far as applicable, based on the available data.

## 3 Results

A total of 55 patients received iFDPs and reached baseline, with 28 (50.9%) allocated to the monolithic zirconia (MonoZR) group and 27 (49.1%) to the porcelain‐fused‐to‐metal (PFM) group. Baseline demographic and clinical characteristics of the study participants according to treatment group are presented in Table 1.

**TABLE 1:** Baseline demographic and clinical characteristics of the study participants according to treatment group.

Characteristic	MonoZR (*n* = 28)	PFM (*n* = 27)
Age, median (Q1–Q3), years	66 (52–72)	69 (56.8–71)
Female, *n* (%)	13 (46.4)	16 (59.3)
Male, *n* (%)	15 (53.6)	11 (40.7)
Maxilla, *n* (%)	13 (46.4)	13 (48.1)
Mandible, *n* (%)	15 (53.6)	14 (51.9)
Free‐end span, *n* (%)	13 (46.4)	10 (38.5)
Bounded span, *n* (%)	15 (53.6)	16 (61.5)
Conventional impression, *n* (%)	23 (82.1)	20 (74.1)
Digital impression, *n* (%)	5 (17.9)	7 (25.9)

*Note:* Smoking status was not systematically recorded; smokers of > 15 cigarettes/day were excluded according to the study protocol. Implant‐level variables (implant placement timing, bone defects, and augmentation procedures) are presented separately in Table 2 because each participant received two implants, and these characteristics could differ between the mesial and distal implant sites.

At the 1‐year follow‐up, 48 patients were available for clinical evaluation (24 per group). Between the 1‐ and 3‐year follow‐up visits, one patient in the PFM group who had previously been lost to follow‐up returned for assessment, two patients (one in each group) experienced implant loss resulting in prosthesis failure, and two additional patients (one per group) were lost to follow‐up.

At the 3‐year follow‐up, 45 patients were available for clinical evaluation (22 in the MonoZR group and 23 in the PFM group).

Free‐end edentulous situations at baseline were present in 23 patients (41.8%), including 13 patients (46.4%) in the MonoZR group and 10 patients (38.5%) in the PFM group, whereas bounded edentulous spans were observed in 15 patients (53.6%) and 16 patients (61.5%), respectively. Implant placement was most frequently performed following a Type 4 timing protocol at both mesial and distal sites. Bone augmentation procedures, including guided bone regeneration and sinus floor elevation, were performed in a subset of cases. Peri‐implant defects requiring augmentation procedures were observed at 23 mesial and 16 distal implant sites (Table 2). Detailed information on implant placement timing and augmentation procedures is provided in Table 2. The CONSORT 2010 flow diagram is presented in Figure 1.

**TABLE 2:** Implant placement timing and bone defect characteristics at baseline.

Parameter	Category	Mesial implant (premolar position) *n* (%)	Distal implant (molar position) *n* (%)
Placement timing	Type 2	6 (10.9%)	1 (1.9%)
	Type 3	8 (14.5%)	5 (9.4%)
	Type 4	41 (74.5%)	47 (88.7%)
Bone defects	None	25 (52.1%)	27 (62.8%)
	Dehiscence	15 (31.3%)	5 (11.6%)
	Fenestration	3 (6.3%)	—
	Sinus	—	9 (20.9%)
	Other	5 (10.4%)	2 (4.7%)
Bone augmentation	GBR	15 (75.0%)	4 (25.0%)
	Sinus lateral	—	7 (43.8%)
	Sinus summers	—	4 (25.0%)
	Contour augmentation	4 (20.0%)	—
	Yxoss	1 (5.0%)	1 (6.3%)

**FIGURE 1:**
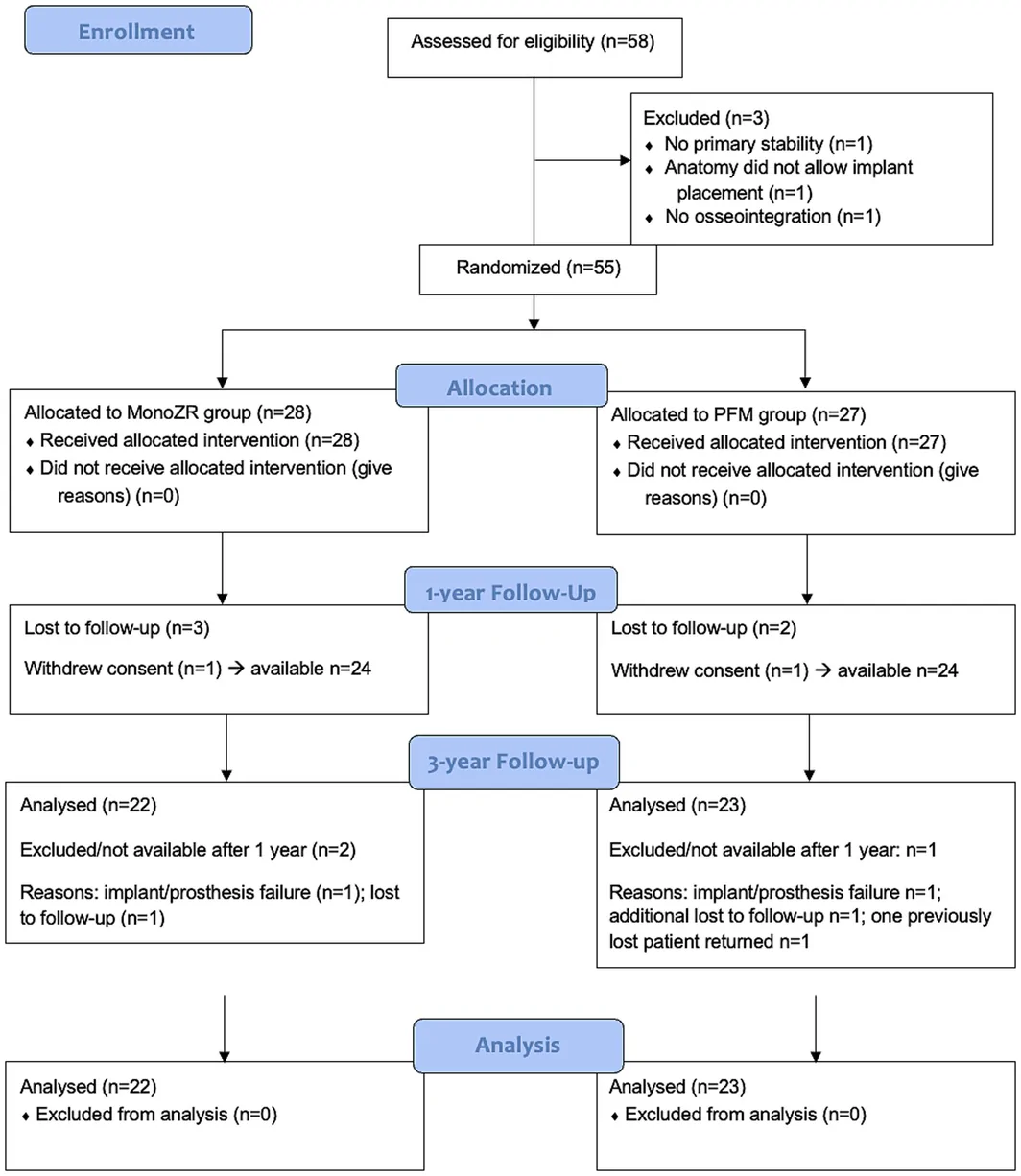
CONSORT 2010 flow diagram.

### 3.1 Survival Outcomes

During the 3‐year follow‐up period, a total of two prosthesis failures were recorded, corresponding to one event in each group. Both failures were associated with implant loss, resulting in loss of the corresponding iFDPs.

Kaplan–Meier analysis demonstrated no statistically significant difference in prosthesis survival between the MonoZR and PFM groups (log‐rank test, *p* = 1.00). Overall prosthesis survival remained high in both groups throughout the observation period.

### 3.2 Technical Complications

Chipping of the veneering material was observed exclusively in the PFM group, affecting 3 of 27 prostheses (11.1%). Two events occurred within the first year of function, while one additional event was recorded between the first and third year of follow‐up. No chipping events were observed in the MonoZR group (0/28). This difference did not reach statistical significance (Fisher's exact test, *p* = 0.11).

### 3.3 Complication‐Free Survival

A total of 11 complications were recorded during the follow‐up period, with 7 events in the PFM group and 4 in the MonoZR group. Kaplan–Meier analysis of complication‐free survival revealed no statistically significant difference between groups (log‐rank test, *p* = 0.40).

### 3.4 Peri‐Implant Clinical Parameters

Descriptive statistics for peri‐implant clinical parameters are presented in Table 3. Linear mixed‐effects analysis revealed no significant differences between the MonoZR and PFM groups for any of the evaluated parameters (all β estimates close to zero, all *p* > 0.05; Tables S1–S3).

**TABLE 3:** Peri‐implant clinical parameters over time (mean ± SD).

Parameter	Time	MonoZR, Mean ± SD	PFM, Mean ± SD
PI	Baseline	0.02 ± 0.05	0.05 ± 0.09
	1 year	0.04 ± 0.08	0.12 ± 0.15
	3 years	0.11 ± 0.18	0.12 ± 0.22
	Baseline	0.21 ± 0.17	0.14 ± 0.16
	1 year	0.29 ± 0.16	0.29 ± 0.18
	3 years	0.40 ± 0.24	0.27 ± 0.20
PD (mm)	Baseline	3.17 ± 0.58	3.00 ± 0.48
	1 year	3.21 ± 0.52	3.10 ± 0.62
	3 years	3.27 ± 0.67	3.10 ± 0.53

Over time, PI values increased significantly at the 3‐year follow‐up compared to baseline (*β* = 0.09, 95% CI: 0.01 to 0.16, *p* = 0.031), whereas no significant change was observed at 1 year (*β* = 0.01, 95% CI: −0.06 to 0.09, *p* = 0.706). Similarly, BoP increased significantly at 3 years (*β* = 0.19, 95% CI: 0.09 to 0.28, *p* < 0.001), with no significant differences at 1 year (*β* = 0.07, 95% CI: −0.02 to 0.17, *p* = 0.123). In contrast, PD remained stable over time, with no statistically significant changes observed at either follow‐up interval (1 year: *β* = 0.04, 95% CI: −0.20 to 0.27, *p* = 0.745; 3 years: *β* = 0.09, 95% CI: −0.15 to 0.34, *p* = 0.442).

No significant group × time interactions were identified for any parameter (all *p* > 0.05; Tables S1–S3), indicating comparable temporal trends between the two groups.

### 3.5 MBL Changes

Descriptive statistics for MBLs at mesial and distal implant sites are presented in Table 4. Linear mixed‐effects analysis demonstrated no significant treatment‐group effect on MBL (*β* = 0.16, 95% CI: −0.23 to 0.55, *p* = 0.427).

**TABLE 4:** Marginal bone levels (MBL) at mesial and distal implant sites over time.

Site	Time	Mean ± SD	Mean ± SD
Mesial	Baseline	−0.31 ± 0.68	−0.48 ± 0.68
Mesial	1 year	−0.62 ± 0.74	−0.55 ± 0.81
Mesial	3 years	−0.57 ± 1.04	−0.35 ± 0.42
Distal	Baseline	−0.31 ± 0.74	−0.61 ± 0.76
Distal	1 year	−0.72 ± 0.58	−0.61 ± 0.69
Distal	3 years	−0.68 ± 0.60	−0.82 ± 0.75

*Note:* Values are presented as mean ± standard deviation.

MBLs remained stable over time, with no significant changes at either 1 year (β = −0.09, 95% CI: −0.41 to 0.23, *p* = 0.573) or 3 years (*β* = 0.11, 95% CI: −0.21 to 0.43, *p* = 0.499). Likewise, no significant effect of implant site was observed (*β* = −0.13, 95% CI: −0.44 to 0.18, *p* = 0.419), and no significant group × time × site interactions were identified (all *p* > 0.05; Table S4), indicating consistent MBL behavior across materials, time points, and implant sites. An additional linear mixed‐effects analysis including bone augmentation as a covariate demonstrated no significant association between bone augmentation procedures and MBL (*β* = 0.15, 95% CI: −0.03 to 0.34, *p* = 0.107; Table S4).

### 3.6 Clinical Performance (USPHS Outcomes)

The clinical performance of the iFDPs, assessed using USPHS criteria, demonstrated high overall success rates in both groups over the 3‐year follow‐up period (Table 5).

**TABLE 5:** Clinical performance of iFDPs according to modified USPHS criteria.

Time point	Criterion	MonoZR *n* (%)	PFM *n* (%)
Baseline	USPHS 1 (Satisfaction)	A: 25 (89.3%), B: 3 (10.7%)	A: 24 (92.3%), B: 2 (7.7%)
	USPHS 4 (Fracture)	A: 28 (100%)	A: 26 (100%)
	USPHS 13 (Wear)	A: 28 (100%)	A: 26 (100%)
1 year	USPHS 1	A: 23 (95.8%), B: 1 (4.2%)	A: 23 (95.8%), B: 1 (4.2%)
	USPHS 4	A: 24 (100%)	A: 22 (91.7%), B: 2 (8.3%)
	USPHS 13	A: 22 (95.7%), B: 1 (4.3%)	A: 22 (91.7%), B: 2 (8.3%)
3 years	USPHS 1	A: 21 (100%)	A: 22 (95.65%), B: 1 (4.35%)
	USPHS 4	A: 21 (100%)	A: 20 (87%), B: 3 (13%)
	USPHS 13	A: 20 (95.2%), B: 1 (4.8%)	A: 18 (78.26%), B: 5 (21.74%)

At baseline, all restorations were rated as Alpha for USPHS criteria 4 (fracture) and 13 (occlusal wear), while minor deviations (Bravo ratings) were observed for USPHS 1 (patient satisfaction).

At the 1‐year follow‐up, the majority of restorations remained within the Alpha category across all evaluated criteria. In the PFM group, some Bravo ratings were observed for veneering fracture (USPHS 4) and occlusal wear (USPHS 13), affecting 8.3% of restorations.

At 3 years, all restorations in the MonoZR group were rated as Alpha for USPHS 1 and all but one for the PFM group (95.65%). While the MonoZR group maintained exclusively Alpha ratings for USPHS 4, the PFM group exhibited an increased proportion of Bravo scores (13%). Similarly, for USPHS 13, Bravo ratings were more frequent in the PFM group (21.74%) compared to the MonoZR group (4.8%).

Between‐group comparisons of USPHS ratings at each follow‐up time point revealed no statistically significant differences (all *p* ≥ 0.345). When dichotomized into clinically acceptable (Alpha/Bravo) and unacceptable (Charlie/Delta) outcomes, all restorations in both groups were classified as acceptable throughout the observation period.

### 3.7 Model Diagnostics

Model assumptions for the linear mixed‐effects analyses were evaluated through inspection of residuals and Q–Q plots, assessment of homoscedasticity, and identification of influential observations using graphical diagnostics. No major violations of model assumptions were detected.

Representative clinical and radiographic cases from the MonoZR and PFM groups are presented in Figures 2 and 3.

**FIGURE 2:**
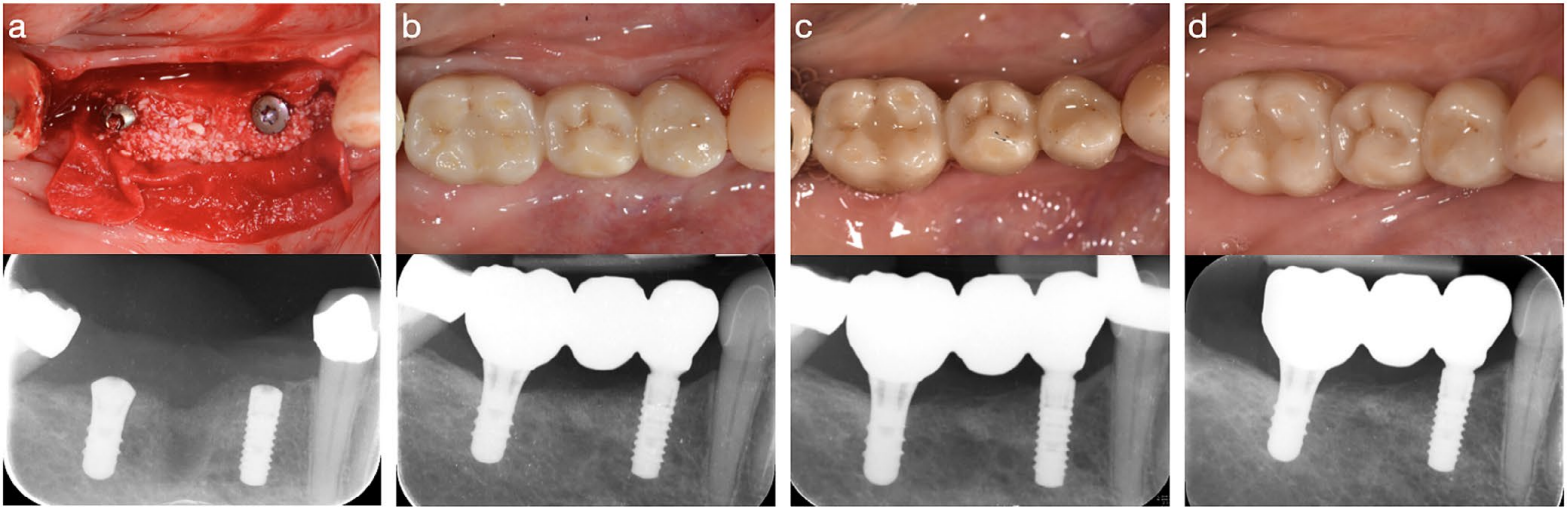
Representative clinical and radiographic case from the MonoZR group. Images show the surgical situation (a), baseline at iFDP insertion (b), 1‐year follow‐up (c), and (d) 3‐year follow‐up. For each time point, the clinical photograph is shown above the corresponding periapical radiograph.

**FIGURE 3:**
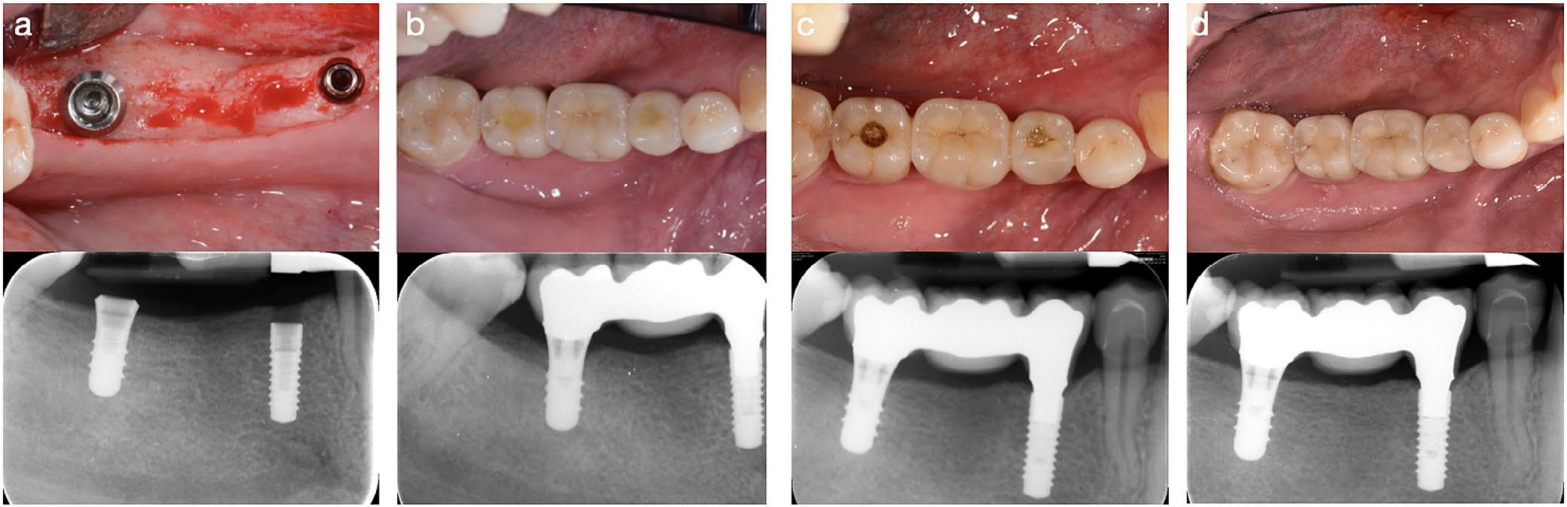
Representative clinical and radiographic case from the PFM group. Images show the surgical situation (a), baseline at iFDP insertion (b), 1‐year follow‐up (c), and (d) 3‐year follow‐up. For each time point, the clinical photograph is shown above the corresponding periapical radiograph.

## 4 Discussion

The present RCT evaluated the 3‐year outcomes of three‐unit iFDPs fabricated from MonoZR and PFM in posterior regions. Overall, both treatment modalities demonstrated high prosthesis survival, stable peri‐implant conditions, and favorable clinical performance. No statistically significant differences were observed between groups for any of the evaluated outcomes. With respect to the primary outcome, no ceramic fractures were recorded in the MonoZR group, whereas veneering ceramic chipping occurred exclusively in the PFM group. Although this difference did not reach statistical significance, the observed pattern is clinically relevant and consistent with the underlying material concepts.

Veneering ceramic chipping remains one of the most frequently reported technical complications in implant‐supported restorations. Systematic reviews have consistently identified veneering fracture as the predominant technical complication in bilayered restorations (Pjetursson et al. 2012; Pieralli et al. 2018; Sailer, Balmer, et al. 2018). The occurrence of chipping exclusively in the PFM group in the present study is in line with these findings.

The absence of chipping in the MonoZR group may be explained by the elimination of the framework–veneer interface, which represents a known weak point susceptible to cohesive and adhesive failures under functional loading (Alsarani et al. 2024). This is particularly relevant in posterior regions, where occlusal forces are increased. Additionally, for PFM restorations, the chipping rate observed in the present study is within the range reported in the literature, further supporting the external validity of the findings (Pjetursson et al. 2012). These findings are also in line with previous studies evaluating monolithic zirconia and PFM restorations in single implant‐supported crowns (Zumstein et al. 2023) and tooth‐supported prostheses (Pjetursson et al. 2021; Pjetursson et al. 2026), where similar survival rates and a reduced incidence of veneering‐related complications have been reported.

In addition to veneering‐related events, the overall frequency of technical and biological complications was low and comparable between groups. Most complications were minor, such as minimal occlusal wear, and could be managed without the need for prosthesis replacement, reflecting favorable clinical performance of both restorative concepts. These findings are aligned with previous studies indicating that complications in iFDPs are often limited in severity and can be effectively managed (Pjetursson et al. 2012; Wittneben et al. 2014; Sailer, Balmer, et al. 2018; da Silva Bezerra et al. 2025). The absence of differences between groups suggests that, within the present follow‐up period, the restorative material did not significantly influence the overall complication profile.

Prosthesis survival was high in both groups, with only two failures recorded during the 3‐year follow‐up period, each associated with implant loss. No statistically significant differences in survival were observed between groups. These findings are in agreement with previous systematic reviews reporting high survival rates for iFDPs, irrespective of material type, over 3 years of function (Pjetursson et al. 2012; Pieralli et al. 2018; Sailer, Balmer, et al. 2018; Pjetursson et al. 2021). Importantly, prosthesis failure in the present study was not related to material‐specific complications but rather to implant loss, highlighting the multifactorial nature of treatment outcomes in implant prosthodontics.

Peri‐implant clinical parameters and MBLs did not differ significantly between groups at any time point. While PD and MBLs remained stable throughout the observation period, PI and BoP increased significantly at the 3‐year follow‐up. These findings suggest that both restorative materials are associated with stable peri‐implant tissue conditions over time, with no material‐related differences observed. The increase in PI and BoP, in the absence of corresponding changes in PD or MBLs, may reflect patient‐related factors, such as oral hygiene, rather than material‐dependent effects (Berglundh et al. 2018). Similar observations have been reported in previous studies on implant‐supported restorations, where peri‐implant tissue conditions and marginal bone stability were not significantly influenced by the prosthetic material (Pjetursson et al. 2012; Zhang et al. 2024; Durrani et al. 2025).

From a clinical perspective, both MonoZR and PFM iFDPs demonstrated favorable medium‐term outcomes. However, the observed trend toward fewer minor technical complications and more stable prosthetic performance with monolithic zirconia may be of clinical importance, particularly in reducing maintenance needs and improving long‐term predictability. The findings suggest that while both materials are viable treatment options, monolithic zirconia may offer advantages in terms of technical reliability, especially in posterior regions subjected to higher occlusal loads. Also, bridge abutments were used at both implant sites as part of a standardized restorative protocol; therefore, caution is warranted when extrapolating the findings to alternative abutment configurations. In addition, although the present study was not designed to compare conventional and digital impression workflows, all restorations achieved clinically acceptable fit at delivery without requiring prosthesis remakes, irrespective of the impression technique used.

A major strength of this study is its randomized controlled design, allowing for direct comparison between two clinically relevant material concepts while minimizing selection bias. Furthermore, standardized surgical and prosthetic protocols, along with calibrated outcome assessment, enhance the internal validity of the study.

However, several limitations should be acknowledged. Firstly, the relatively small sample size and low event rate may have limited the ability to detect statistically significant differences between groups. Secondly, the high overall success rates resulted in a ceiling effect for dichotomized outcomes, reducing the sensitivity of certain analyses. Thirdly, the 3‐year follow‐up provides valuable information on the medium‐term clinical performance of the restorations; however, longer follow‐up is required to assess long‐term outcomes and complications associated with prosthetic materials. Smoking status was not systematically documented. Also, blinding of examiners was not feasible due to the inherent differences between materials, although objective outcome measures were applied where possible.

Given the medium‐term nature of the present findings, longer follow‐up periods are required to confirm the durability of these outcomes. Future studies with larger sample sizes and extended observation periods are needed to further evaluate long‐term complication patterns and to support evidence‐based material selection in implant prosthodontics.

## 5 Conclusion

Both MonoZR and PFM three‐unit posterior iFDPs demonstrated high survival and favorable biological outcomes after 3 years. MonoZR was associated with an absence of chipping events, whereas veneering ceramic chipping occurred only in the PFM group. Although no statistically significant differences were found between materials, monolithic zirconia appears to be a promising alternative to PFM for posterior implant‐supported restorations in the mid‐term.

## Author Contributions

**Ronald E. Jung:** investigation, supervision, resources, writing – review and editing. **Aspasia Pachiou:** methodology, software, data curation, formal analysis, visualization, writing – original draft. **Marc Balmer:** investigation, validation, writing – review and editing. **Alexis Ioannidis:** methodology, investigation, supervision, funding acquisition, project administration, writing – review and editing. **Raphael Singh:** formal analysis, data curation, writing – original draft. **Goran I. Benic:** conceptualization, investigation, funding acquisition, resources, writing – review and editing.

## Funding

This study was supported by an unrestricted grant from ITI Foundation (ITI Grant No. 1030_2014). Study materials such as implants and abutments were provided by Institute Straumann AG.

## Ethics Statement

Approved by the local ethics committee of the Canton of Zurich, Switzerland (Ref. BASEC‐Nr. 2016‐00840).

## Consent

Written informed consent was obtained from all participants prior to inclusion in the study.

## Conflicts of Interest

All authors have provided separate conflicts of interest declarations. R.J., G.B. provided lectures or consultations, which were reimbursed by Institute Straumann AG.

## Supporting information


**Table S1:** Fixed‐effect estimates from the linear mixed‐effects models for Plaque Index.
**Table S2:** Fixed‐effect estimates from the linear mixed‐effects models for Bleeding on Probing.
**Table S3:** Fixed‐effect estimates from the linear mixed‐effects models for Probing Depth.
**Table S4:** Fixed‐effect estimates from the linear mixed‐effects models for Marginal Bone Loss.

**Data S1:** Supporting Information

## Data Availability

The datasets generated and/or analyzed during the current study are not publicly available due to patient confidentiality and institutional data protection regulations but are available from the corresponding author upon reasonable request.

## References

[clr70160-bib-0029] Ainamo, J. , and I. Bay . 1975. “Problems and proposals for recording gingivitis and plaque.” International dental journal 25, no. 4: 229–235. 1058834

[clr70160-bib-0001] Alsarani, M. M. , O. Y. El‐Mowaf , T. W. Coyle , A. Rizkalla , and J. Fava . 2024. “Stress Distribution of Monolithic and Veneered Three‐Unit Zirconia FDPS: A Finite Element Analysis.” International Journal of Prosthodontics, no. 3: 112–122. 10.11607/ijp.8248. 37196037

[clr70160-bib-0002] Berglundh, T. , G. Armitage , M. G. Araujo , et al. 2018. “Peri‐Implant Diseases and Conditions: Consensus Report of Workgroup 4 of the 2017 World Workshop on the Classification of Periodontal and Peri‐Implant Diseases and Conditions.” Journal of Clinical Periodontology 45, no. Suppl 20: S286–S291. 10.1111/jcpe.12957 29926491

[clr70160-bib-0003] Chen, S. T. , T. G. Wilson , and C. H. F. Hämmerle . 2004. “Immediate or Early Placement of Implants Following Tooth Extraction: Review of Biologic Basis, Clinical Procedures, and Outcomes.” International Journal of Oral & Maxillofacial Implants 19: 12–25. 15635942

[clr70160-bib-0004] Cheng, C.‐W. , C.‐H. Chien , C.‐J. Chen , and P. Papaspyridakos . 2018. “Clinical Results and Technical Complications of Posterior Implant‐Supported Modified Monolithic Zirconia Single Crowns and Short‐Span Fixed Dental Prostheses: A 2‐Year Pilot Study.” Journal of Prosthodontics: Official Journal of the American College of Prosthodontists 27: 108–114. 10.1111/jopr.12682 29086467

[clr70160-bib-0005] Cheng, C.‐W. , C.‐H. Chien , C.‐J. Chen , and P. Papaspyridakos . 2019. “Randomized Controlled Clinical Trial to Compare Posterior Implant‐Supported Modified Monolithic Zirconia and Metal‐Ceramic Single Crowns: One‐Year Results.” Journal of Prosthodontics 28: 15–21. 10.1111/jopr.12767 29528175

[clr70160-bib-0006] da Silva Bezerra, A. , A. K. C. Ribeiro , A. H. Veríssimo , and E. O. de Almeida . 2025. “Prosthetic Complications and Failures of Implant‐Supported Fixed Partial Dentures: A Scoping Review.” Journal of Prosthetic Dentistry 134: 643–650. 10.1016/j.prosdent.2023.10.025 38036319

[clr70160-bib-0007] Durrani, F. , A. Pandey , R. Muralisekar , S. Agarwal , S. Shilpi , and K. K. Rajak . 2025. “Periodontal and Prosthetic Perspective of Implant‐Supported Full‐Arch Prostheses With Monolithic Zirconia and Porcelain Fused Metal Restorative Materials: A Three‐Year Retrospective Case Series.” Journal of Indian Society of Periodontology 29: 318–324. 10.4103/jisp.jisp_415_23 PMC12677748 41357450

[clr70160-bib-0008] Faul, F. , E. Erdfelder , A.‐G. Lang , and A. Buchner . 2007. “G*Power 3: A Flexible Statistical Power Analysis Program for the Social, Behavioral, and Biomedical Sciences.” Behavior Research Methods 39: 175–191. 10.3758/bf03193146 17695343

[clr70160-bib-0009] Gallucci, G. O. , A. Hamilton , W. Zhou , D. Buser , and S. Chen . 2018. “Implant Placement and Loading Protocols in Partially Edentulous Patients: A Systematic Review.” Clinical Oral Implants Research 29, no. Suppl. 16: 106–134. 10.1111/clr.13276 30328194

[clr70160-bib-0010] Heintze, S. D. , and V. Rousson . 2010. “Survival of Zirconia‐ and Metal‐Supported Fixed Dental Prostheses: A Systematic Review.” International Journal of Prosthodontics 23: 493–502. 21209982

[clr70160-bib-0011] Hjerppe, J. , A. Ioannidis , A. M. Valdés , et al. 2025. “Posterior Monolithic 3‐Unit Zirconia Fixed Dental Prostheses: One‐Year Interim Results of a Multicenter Randomized Clinical Trial.” Journal of Dentistry 160: 105900. 10.1016/j.jdent.2025.105900 40505806

[clr70160-bib-0012] Ioannidis, A. , M. Balmer , K. Pala , A. Mehl , and A. Bindl . 2026. “5‐Year Clinical Outcomes of Three‐Unit Posterior Zirconia‐ Ceramic Fixed Dental Prostheses: A Randomized Trial Comparing Manually and CAD/CAM Layered Veneering.” International Journal of Computerized Dentistry 0: 0. 10.3290/j.ijcd.b6951842 41801291

[clr70160-bib-0013] Johansson, C. , G. Kmet , J. Rivera , C. Larsson , and P. Vult Von Steyern . 2014. “Fracture Strength of Monolithic All‐Ceramic Crowns Made of High Translucent Yttrium Oxide‐Stabilized Zirconium Dioxide Compared to Porcelain‐Veneered Crowns and Lithium Disilicate Crowns.” Acta Odontologica Scandinavica 72: 145–153. 10.3109/00016357.2013.822098 23865549

[clr70160-bib-0014] Kim, W. , X. C. Li , and A. S. Bidra . 2023. “Clinical Outcomes of Implant‐Supported Monolithic Zirconia Crowns and Fixed Partial Dentures: A Systematic Review.” Journal of Prosthodontics: Official Journal of the American College of Prosthodontists 32: 102–107. 10.1111/jopr.13575 35929416

[clr70160-bib-0015] Koenig, V. , A. J. Vanheusden , S. O. Le Goff , and A. K. Mainjot . 2013. “Clinical Risk Factors Related to Failures With Zirconia‐Based Restorations: An up to 9‐Year Retrospective Study.” Journal of Dentistry 41: 1164–1174. 10.1016/j.jdent.2013.10.009 24135294

[clr70160-bib-0030] O'Leary, T. J. , R. B., Drake , and J. E. Naylor . 1972. “The plaque control record.” Journal of periodontology 43, no. 1: 38. 10.1902/jop.1972.43.1.38. 4500182

[clr70160-bib-0016] Pieralli, S. , R.‐J. Kohal , K. Rabel , M. von Stein‐Lausnitz , K. Vach , and B. C. Spies . 2018. “Clinical Outcomes of Partial and Full‐Arch All‐Ceramic Implant‐Supported Fixed Dental Prostheses. A Systematic Review and Meta‐Analysis.” Clinical Oral Implants Research 29, no. Suppl 18: 224–236. 10.1111/clr.13345 30306694

[clr70160-bib-0017] Pjetursson, B. E. , V. Fehmer , and I. Sailer . 2022. “EAO Position Paper: Material Selection for Implant‐Supported Restorations.” International Journal of Prosthodontics 35: 7–16. 10.11607/ijp.8013 35230351

[clr70160-bib-0018] Pjetursson, B. E. , J. Pitta , A. Balet , G. R. Bjarnadottir , I. Sailer , and P. Romandini . 2026. “A Systematic Review and Meta‐Analysis Evaluating the Survival, the Failure and the Complication Rates of Metal‐Ceramic, Veneered and Monolithic All‐Ceramic Tooth‐Supported Single Crowns.” International Journal of Prosthodontics 39: 308. 10.11607/ijp.9633 41489982

[clr70160-bib-0019] Pjetursson, B. E. , I. Sailer , A. Latyshev , K. Rabel , R.‐J. Kohal , and D. Karasan . 2021. “A Systematic Review and Meta‐Analysis Evaluating the Survival, the Failure, and the Complication Rates of Veneered and Monolithic All‐Ceramic Implant‐Supported Single Crowns.” Clinical Oral Implants Research 32, no. Suppl 21: 254–288. 10.1111/clr.13863 PMC9293296 34642991

[clr70160-bib-0020] Pjetursson, B. E. , D. Thoma , R. Jung , M. Zwahlen , and A. Zembic . 2012. “A Systematic Review of the Survival and Complication Rates of Implant‐Supported Fixed Dental Prostheses (FDPs) After a Mean Observation Period of at Least 5 Years.” Clinical Oral Implants Research 23, no. Suppl 6: 22–38. 10.1111/j.1600-0501.2012.02546.x 23062125

[clr70160-bib-0021] Rabel, K. , B. C. Spies , S. Pieralli , K. Vach , and R.‐J. Kohal . 2018. “The Clinical Performance of All‐Ceramic Implant‐Supported Single Crowns: A Systematic Review and Meta‐Analysis.” Clinical Oral Implants Research 29, no. Suppl 18: 196–223. 10.1111/clr.13337 30306684

[clr70160-bib-0022] Romandini, P. , B. E. Pjetursson , J. Pitta , A. Balet , R. Ikumi , and I. Sailer . 2026. “A Systematic Review and Meta‐Analysis Evaluating the Survival, the Failure and the Complication Rates of Metal‐Ceramic, Veneered and Monolithic All‐Ceramic Tooth‐Supported Multiple‐ Unit Fixed Dental Prostheses (Fdps).” International Journal of Prosthodontics 39, no. 4: 444–462. 10.11607/ijp.9666. 41650383

[clr70160-bib-0023] Sailer, I. , M. Balmer , J. Hüsler , C. H. F. Hämmerle , S. Känel , and D. S. Thoma . 2018. “10‐Year Randomized Trial (RCT) of Zirconia‐Ceramic and Metal‐Ceramic Fixed Dental Prostheses.” Journal of Dentistry 76: 32–39. 10.1016/j.jdent.2018.05.015 29807060

[clr70160-bib-0024] Sailer, I. , M. Strasding , N. A. Valente , M. Zwahlen , S. Liu , and B. E. Pjetursson . 2018. “A Systematic Review of the Survival and Complication Rates of Zirconia‐Ceramic and Metal‐Ceramic Multiple‐Unit Fixed Dental Prostheses.” Clinical Oral Implants Research 29, no. Suppl 16: 184–198. 10.1111/clr.13277 30328185

[clr70160-bib-0025] Strasding, M. , L. Marchand , E. Merino , C. Zarauz , and J. Pitta . 2024. “Material and Abutment Selection for CAD/CAM Implant‐Supported Fixed Dental Prostheses in Partially Edentulous Patients ‐ A Narrative Review.” Clinical Oral Implants Research 35: 984–999. 10.1111/clr.14315 38864592

[clr70160-bib-0026] Wittneben, J.‐G. , D. Buser , G. E. Salvi , W. Bürgin , S. Hicklin , and U. Brägger . 2014. “Complication and Failure Rates With Implant‐Supported Fixed Dental Prostheses and Single Crowns: A 10‐Year Retrospective Study.” Clinical Implant Dentistry and Related Research 16: 356–364. 10.1111/cid.12066 23551688

[clr70160-bib-0027] Zhang, Y. , J. Luo , P. Di , et al. 2024. “Screw‐Retained Ceramic‐Veneered/Monolithic Zirconia Partial Implant‐Supported Fixed Dental Prostheses: A 5 to 10‐Year Retrospective Study on Survival and Complications.” Journal of Prosthodontics: Official Journal of the American College of Prosthodontists 33: 221–230. 10.1111/jopr.13723 37302066

[clr70160-bib-0028] Zumstein, K. , T. Waller , C. H. F. Hämmerle , R. E. Jung , G. Benic , and S. Mühlemann . 2023. “Clinical Performance of Monolithic Zirconia Crowns on Titanium‐Zirconium Reduced‐Diameter Implants in the Molar Area: Interim Data at Three Years of a Randomized Controlled Trial.” Clinical Oral Implants Research 34: 1354–1362. 10.1111/clr.14179 37668109

