# Supplementary material for: Monolithic Zirconia Versus Porcelain‐Fused‐to‐Metal Implant‐Supported Fixed Dental Prostheses in the Posterior Region: 3‐Year Results of a Randomized Controlled Clinical Trial
Source: Clin Oral Implants Res. 2026 Aug 21;37(10):1197–207. doi: 10.1111/clr.70160 (PMC13644546; doi:10.1111/clr.70160)
Supplement: Supplementary file 1 [file CLR-37-1197-s001.doc]

**Supplementary Tables**

**A. Plaque Index**

| **Predictor** | **β** | **95% CI** | ***p*** |
| --- | --- | --- | --- |
| Group | 0.03 | −0.05 to 0.10 | 0.458 |
| Time (1 year) | 0.01 | −0.06 to 0.09 | 0.706 |
| Time (3 years) | **0.09** | **0.01 to 0.16** | **0.031** |
| Group × Time (1 year) | 0.06 | −0.05 to 0.16 | 0.310 |
| Group × Time (3 years) | −0.02 | −0.13 to 0.09 | 0.702 |

**Supplementary Table S1. Fixed-effect estimates from the linear mixed-effects models for Plaque Index.**

**B. Bleeding on Probing**

| **Predictor** | **β** | **95% CI** | ***p*** |
| --- | --- | --- | --- |
| Group | −0.07 | −0.17 to 0.03 | 0.146 |
| Time (1 year) | 0.07 | −0.02 to 0.17 | 0.123 |
| Time (3 years) | **0.19** | **0.09 to 0.28** | **<0.001** |
| Group × Time (1 year) | 0.08 | −0.06 to 0.21 | 0.250 |
| Group × Time (3 years) | −0.05 | −0.18 to 0.09 | 0.503 |

**Supplementary Table S2. Fixed-effect estimates from the linear mixed-effects models for Bleeding on Probing.**

**C. Probing Depth**

| **Predictor** | **β** | **95% CI** | ***p*** |
| --- | --- | --- | --- |
| Group | −0.17 | −0.48 to 0.13 | 0.266 |
| Time (1 year) | 0.04 | −0.20 to 0.27 | 0.745 |
| Time (3 years) | 0.09 | −0.15 to 0.34 | 0.442 |
| Group × Time (1 year) | 0.07 | −0.26 to 0.40 | 0.679 |
| Group × Time (3 years) | 0.02 | −0.32 to 0.36 | 0.910 |

**Supplementary Table S3. Fixed-effect estimates from the linear mixed-effects models for Probing Depth.**

**D. Marginal Bone Loss**

| **Predictor** | **β** | **95% CI** | ***p* value** |
| --- | --- | --- | --- |
| Group (MonoZR vs PFM) | 0.16 | −0.23 to 0.55 | 0.427 |
| Time (1 year vs baseline) | −0.09 | −0.41 to 0.23 | 0.573 |
| Time (3 years vs baseline) | 0.11 | −0.21 to 0.43 | 0.499 |
| Implant site (Distal vs Mesial) | −0.13 | −0.44 to 0.18 | 0.419 |
| Group × Time (1 year) | −0.17 | −0.61 to 0.28 | 0.459 |
| Group × Time (3 years) | −0.34 | −0.79 to 0.12 | 0.143 |
| Group × Implant site | 0.12 | −0.31 to 0.56 | 0.578 |
| Time (1 year) × Implant site | 0.06 | −0.38 to 0.51 | 0.777 |
| Time (3 years) × Implant site | −0.34 | −0.79 to 0.11 | 0.139 |
| Group × Time (1 year) × Implant site | −0.16 | −0.78 to 0.47 | 0.618 |
| Group × Time (3 years) × Implant site | 0.24 | −0.40 to 0.87 | 0.466 |
| Bone augmentation (Yes vs No) | 0.15 | -0.03 to 0.34 | 0.107 |

**Supplementary Table S4. Fixed-effect estimates from the linear mixed-effects models for Marginal Bone Loss.**
